# The Use of Platelet-Rich Plasma for Treatment of Tenodesmic Lesions in Horses: A Systematic Review and Meta-Analysis of Clinical and Experimental Data

**DOI:** 10.3390/ani11030793

**Published:** 2021-03-12

**Authors:** Chiara Montano, Luigi Auletta, Adelaide Greco, Dario Costanza, Pierpaolo Coluccia, Chiara Del Prete, Leonardo Meomartino, Maria Pia Pasolini

**Affiliations:** 1 Veterinary Teaching Hospital, School of Veterinary Medicine, University of Córdoba, 14004 Córdoba, Spain; c.montano@hotmail.it; 2 Institute of Biostructure and Bioimaging, National Research Council (IBB CNR), Via T. De Amicis 95, 80145 Napoli, Italy; luigi.auletta@yahoo.it; 3 Interdepartmental Centre of Veterinary Radiology, University of Napoli “Federico II”, Via Federico Delpino 1, 80137 Napoli, Italy; adegreco@unina.it (A.G.); pierpaolo.coluccia@unina.it (P.C.); meomarti@unina.it (L.M.); 4 Department of Veterinary Medicine and Animal Production, University of Napoli “Federico II”, Via Federico Delpino 1, 80137 Napoli, Italy; chiara.delprete@unina.it (C.D.P.); pasolini@unina.it (M.P.P.)

**Keywords:** DDFT, equine, growth factors, healing, ligament, platelet concentrate, PRP, SDFT, SL, tendon

## Abstract

**Simple Summary:**

Tenodesmic injuries are key problems for sport horses. Several therapies have been developed over the years, including platelet-rich plasma (PRP), an autologous product that should stimulate tissue regeneration with its high concentration of growth factors. Nowadays, there are conflicting reports concerning the effect of PRP in tenodesmic lesions. The aim of our systematic review was to determine the effect of PRP on tendons and ligaments healing through a meta-analysis, a process to determine consensus from across published studies. The meta-analysis is the quantitative component of a systematic review, a statistical synthesis of the published data about a topic. We selected studies that evaluate PRP therapy in vitro or in vivo, most of which had a high risk of bias. The results found there is no evidence that PRP enhances the healing of tendon and ligament injuries. In the future, further unbiased, blinded, and controlled studies are needed to clarify the efficacy of this platelet concentrate in the treatment of equine tendon and ligament injuries.

**Abstract:**

The use of platelet-rich plasma (PRP) to enhance tenodesmic lesion healing has been questioned over the years. The aim of this study was to evaluate current literature to establish the effectiveness of PRP for treating tenodesmic lesions through a systematic review, in accordance with the PRISMA guidelines, and a meta-analysis. Studies comparing PRP with placebo or other treatments for horses with tenodesmic injuries or evaluated PRP effect on tendon and ligament explants were included. Outcomes were clinical, ultrasound, histologic, molecular evaluation, and adverse effects. Two authors independently extracted data and assessed each study’s risk of bias. Treatment effects were evaluated using risk ratios for dichotomous data, together with 95% CI. Data were pooled using the random-effects model. The quality of the evidence for each outcome was assessed using GRADE criteria. Twenty-four trials met inclusion criteria for systematic review, while fifteen studies were included in the meta-analysis. Results showed no significant differences in the outcomes between PRP and control groups. Finally, there is no definitive evidence that PRP enhances tendons and ligaments healing. Therefore, there is a need for more controlled trials to draw a firmer conclusion about the efficacy of PRP as a treatment for tenodesmic lesions in the horse.

## 1. Introduction

Tendon and ligament injuries are a serious career-compromising disease and a major cause of lameness, reduced performance, and premature retirement in horses of every discipline and aptitude [1,2,3]. Tendons tend to heal slowly, forming disorganized and collagen-rich scar tissue, with inferior mechanical properties compared to intact tendons [4]. Thus, reinjury is frequent [5,6], and rational intervention strategies to prevent it is crucial to improve horse welfare and racing career longevity [7]. Over the years, many new conservative and surgical therapies were studied to treat these injuries. However, none of them provide a complete restoration of the anatomical and functional integrity of the injured tissues [1,5,6,8].

Slow healing and mechanically inferior scars have been attributed to the poor vascularization and scarcity in progenitor cells of the tendon tissue [9]. Knowledge concerning tendon repair stimulated research regarding the application of regenerative therapies, aimed to restore the normal structure and biomechanics of the tissues using autologous blood derivatives such as platelet-rich plasma (PRP) and mesenchymal stem cells (MSCs), both in human and veterinary medicine [10,11,12,13,14,15,16,17].

Platelets in PRP can release several growth factors and induce the production of anti-inflammatory cytokines, enhancing hemostasis, promoting the regeneration of tissue cells and stroma through different ways, and accelerating tissue repair [18,19,20]. The biological effects of PRP include an increase in type I collagen production, the proliferation of tenocytes, neovascularization, increased resistance, and a better organization and alignment of the fibers [9]. As PRP mechanism of action is still unclear, preparation standards and methods have not been unified, and its clinical efficacy is still controversial [21].

Clinical case series, experimental animal studies, placebo-controlled studies of naturally occurring injuries, and in vitro and ex vivo studies evaluated the effects of PRP in the horse. Systematic PRP evaluation for repairing tendon and ligament injuries may provide a scientific reference for treatment and clinical application strategies. Indeed, although in human and veterinary medicine several systematic reviews and meta-analyses have been subsequently published [3,15,21,22,23,24,25,26,27,28,29], it is still not clear which product or combination of substrates is most appropriate related to individual cases. Due to the variety of lesions and products, clinical studies would require large numbers, with economic and ethical concerns [30,31,32]. Thus, we decided to include in vitro and ex vivo studies in the meta-analysis, assuming a good standardization of the procedures compared to clinical trials.

The aim of this study was to elucidate this controversial issue, conducting a systematic review and meta-analysis of in vivo and in vitro studies about PRP efficacy to assess the effects (benefits and harms) of platelet-rich therapies for treating tenodesmic injuries in horses.

## 2. Materials and Methods

### 2.1. Criteria for Considering Studies for This Review

The systematic review was performed in accordance with the PRISMA (Preferred Reporting Items for Systematic Reviews and Meta-Analyses) statement [33], while the meta-analysis was performed using the program Review Manager 5.3 (Review Manager (RevMan) (Computer program) Version 5.3. Copenhagen: The Nordic Cochrane Centre, The Cochrane Collaboration, 2014).

The research was carried out between August 2019 and December 2020. A comprehensive literature search addressing PRP use in tendon and ligament injuries in horses was conducted for all the studies published in English, Portuguese, Spanish, and Italian language between 2000 and 2020. Two of the authors (MPP and CM) were involved in the studies’ research. Disagreements were resolved by discussion or by arbitration by a third author (LA).

Search inclusion criteria were the presence of terms such as “PRP”, “platelet-rich plasma”, “tendon”, “ligament”, “horse”, and “equine” in full manuscript, abstract, title, and keywords of publication searched on web search engines that index the full text or metadata of scholarly literature (e.g., Pubmed, Worldcat, Wide Science, Google Scholar, and Scopus).

Studies were excluded from the systematic review and meta-analysis process if they evaluated species other than horses, use another biomaterial and/or substance with PRP, use PRP in lesions different than ligament and tendon injuries.

Because of their scarcity, clinical trials were included independently of their level of evidence or design and without differentiating studies that have a control group from studies that do not have one.

### 2.2. Data Extraction and Management

Two authors (MPP and CM) independently extracted data using a pre-piloted data extraction form. When necessary, a third author (LA) was involved in solving any disagreement. For each trial included in the systematic review, the following data were extracted: year of publication, authors, journal of publication, type of intervention, treated tissue, studies classification such as in vivo or in vitro studies, randomized controlled trials (RCTs) or not (No-RCTs), controlled laboratory study (CLS), sample size, control group, outcome measurements, main results (positive, negative, neutral), adverse events, and bias.

### 2.3. Types of Studies and Participants

We included RCTs, No-RCTs, randomized controlled experimental trials, non-randomized controlled experimental trials, and CLS, comparing platelet-rich therapy with placebo for musculoskeletal soft tissue injuries in adult horses or cultured cells from tendon and ligament explants.

The treatments in the studies were performed in horses affected by injuries of the Superficial Digital Flexor Tendon (SDFT), Deep Digital Flexor Tendon (DDFT), or Suspensory Ligament (SL). Laboratory studies were performed culturing SDFT and SL explants in medium added with PRP.

We place no restrictions in diagnostic methods or criteria used by individual studies, duration of the injury, follow-up period, and evaluation of the outcome.

### 2.4. Types of Interventions

We considered studies where platelet-rich therapies were the only treatment or additional to conservative therapy. Such studies compared platelet-rich therapy with no platelet-rich therapy or placebo. There was no restriction based on the number of procedures or injections and treatment dosage.

### 2.5. Types of Outcome Measures

We categorized the outcome as positive, negative, or neutral effects.

Improvement of the lameness degree and ultrasonographic appearance, return to the same/higher level of competition, high concentration of anti-inflammatory molecules, low concentration of proinflammatory factors, low reinjury rate, and realignment of collagen fibers at the histological examination were considered as positive effects. The opposite ones were negative outputs. The absence of difference in the evaluated outputs between the control group and the treated group or between PRP-group and data obtained from previous studies were neutral effects. First, an overall effect was evaluated, regardless of the outcomes assessed in the study. Second, histologic, ultrasonographic, and biomolecular outcomes examined in multiple studies were separately evaluated. Local and systemic adverse effects of platelet-rich therapy (or placebo) administration (including pain, swelling, infection, and anaphylactic reaction) were recorded.

### 2.6. Measures of Treatment Effect and Assessment of Heterogeneity

Risk ratios with 95% confidence intervals (CI) for dichotomous outcomes were presented.

Heterogeneity between studies was quantified using the I^2^ statistic. An I^2^ value > 75% to indicate high heterogeneity was chosen [34]. Values of *p* < 0.05 were considered significant.

### 2.7. Risk of Bias

The risk of bias of the selected studies was presented according to the PRISMA guidelines [33], and trials were assigned as having a high or low risk of bias. Together with the analysis of the clinical trials and experimental studies’ results, this information was used to verify a possible association between bias and the trial’s output.

## 3. Results

### 3.1. Systematic Review

**Figure 1:**
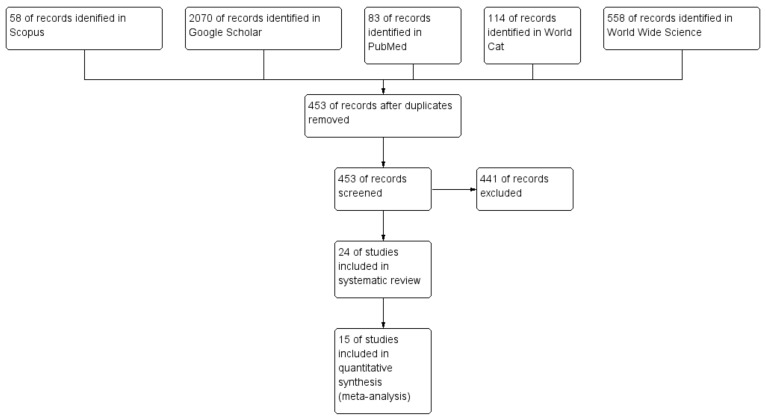
Flowchart for identification of published studies. Review Manager (RevMan 5.3).

Figure 1 shows how the studies were included. A total of 2883 articles were identified after the initial electronic and manual research. Of the 453 articles screened, we selected only 24 that respect the inclusion criteria. Selected studies are reported and numbered in Table 1.

From the selected clinical trials, three were classified as RCTs [3,35,36] (study ID in Table 1: 3, 10, 11); the other seven studies were classified as CLS [31,37,38,39,40,41] (study ID in Table 1: 7, 13, 14, 20–22).

Seven studies [3,31,37,38,39,40,41] (study ID in Table 1: 3, 7, 13, 14, 20–22) of the twenty-four included in this review described in vitro investigations, and the remaining seventeen [30,35,36,42,43,44,45,46,47,48,49,50,51,52,53,54,55] originated from in vivo experiments.

Most of the trials studied the effect of PRP on tendons [30,31,36,37,40,43,44,45,47,48,51,55] (study ID in Table 1: 2, 4–7, 9, 11, 12, 14, 17, 21, 24); fewer articles analyzed its efficacy on ligaments [35,39,40,41,46,50,53,54] (study ID in Table 1: 8, 10, 16, 19, 20–23), and five of them [3,38,42,49,52] (study ID in Table 1: 1, 3, 13, 15, 18) examined its influence in the healing of lesions of both SDFT and SL. Twenty-two out of twenty-four studies reported positive effects by PRP in tendinitis and desmitis treatment, whereas in one study [35] (study ID in Table 1: 10), no difference between control and treated groups was reported. Similarly, Estrada et al. [47] (study ID in Table 1: 9) conclude that the blood-concentrated injected during the proliferative phase of healing in surgically induced superficial digital flexor tendon (SDFT) core lesions have a minor effect on tendon’s healing when ultrasonographic, biochemical, biomechanical, and histological characteristics were compared with the control group.

The most striking feature that affects the quality of the clinical trials included in this review was the lack of a placebo control group in nine of twenty-four studies. The control group (another horse, the contralateral limb, another culture medium, or another treatment) was present in fifteen studies.

A small number of cases’ enrollment was the second most frequent flaw, often highlighted by the same authors as their study’s limitations.

Some studies [23,35,50,54,55] (study ID in Table 1: 10, 11, 16, 23, 24) selected horses from the patients referred to respective hospitals, while other trials do not specify the selection process. The age, sex, breed, and aptitude of the horses selected for the investigations differ in the same study and between different studies.

Most of the trials selected indicate PRP concentration and the methods employed for its preparation, as shown in Table 2.

Regarding PRP activation, three studies [48,54,55] (study ID in Table 1: 12, 23, 24) activated PRP: calcium was used in two studies [48,55] (study ID in Table 1: 12, 24) and trombine in one [54] (study ID in Table 1: 23). In the remaining studies, PRP was not activated. Operators blinding was absent in all studies except one [30] (study ID in Table 1: 4). Animals studied were unaware of the treatment they were subjected to, but the clinicians who administered PRP or placebo almost always knew what they were administering. Garrett et al. [35] (study ID in Table 1: 10) specifies how the operators were aware of what they were injecting into the lesion because PRP and placebo (usually saline solution) have a different appearance.

Concerning the follow-up, all in vivo trials described post-operative care and a rehabilitation program to which every horse was subjected.

To assess the effectiveness of the treatment, trials evaluated different outcomes, as shown in Table 3.

No side effects have ever been reported following PRP administration: two studies [35,48] (study ID in Table 1: 10, 12) described a mild and transient inflammation of the tissues after the injection of the blood concentrate.

#### Risk of Bias of Selected Studies

Table 1 shows the risk of bias of every clinical trial included in the systematic review and meta-analysis. As shown, most of the studies that confirm PRP benefits for the treatment of tenodesmic lesions were associated with a high risk of bias.

### 3.2. Meta-Analysis

Only fifteen of twenty-four trials were included in the meta-analysis [3,30,31,35,36,38,39,40,41,44,45,47,48,51,54]. Studies testing stem cells or other biomaterials together with PRP or without a control group were excluded. Selected studies were reported in bold in Table 3.

Three trials were classified as RCTs [3,35,36] (study ID in Table 1: 3, 10, 11); in vitro studies were all classified as CLS.

The animals selected in the studies were horses of different age, breed, sex, and attitude; a horse treated with saline solution or with another treatment [35,36] (study ID in Table 1: 10, 11), the contralateral limb of the same animal injected with saline solution [30,44,45,47,48,51] (study ID in Table 1: 4–6, 9, 12, 17), tissue explants incubated with a placebo [3,31,38,39,40,41] (study ID in Table 1: 3, 13, 14, 20–22), or other affected horses that did not receive any treatment [54] (study ID in Table 1: 23) were used as control groups.

All the studies highlighted PRP positive influence in the healing of tenodesmic lesions. One trial [35] (study ID in Table 1: 10) reported no difference in the healing process between lesions treated with PRP or saline solution, whereas the study by Estrada et al. [47] (study ID in Table 1: 9) suggested the blood concentrate had limited effects on healing.

Platelet concentrate has been tested on tendons in nine trials [23,30,31,40,44,45,47,48,51] (study ID in Table 1: 4–6, 9, 11, 12, 14, 17, 21), on ligaments in five experiments [35,39,41,54] (study ID in Table 1: 10, 20, 22, 23), and on both structures in two studies [3,38] (study ID in Table 1: 3, 13).

**Figure 2:**
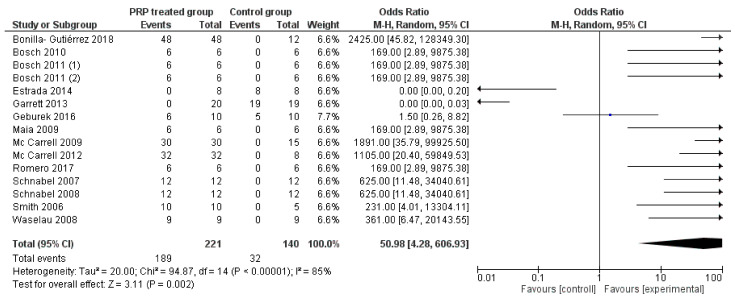
Forest plot representing successful outcomes of selected studies, using a meta-analytic approach based on the random-effects model. The squares represent the positive outcome of individual studies, with the whiskers corresponding to the 95% confidence interval (95% CI). The diamonds correspond to the positive outcome of the study and 95% CI of overall.

**Figure 3:**
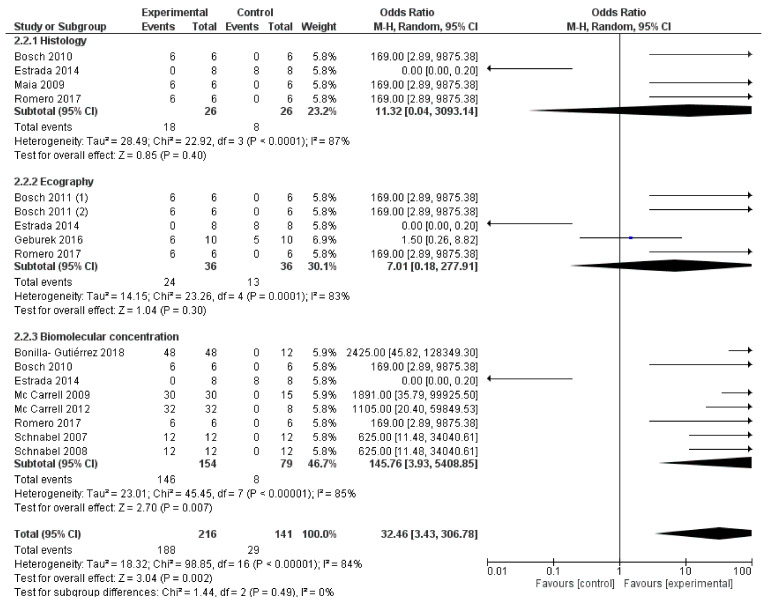
Forest plot representing successful outcomes of selected studies, divided into different subgroups, using a meta-analytic approach based on the random-effects model. The squares represent the positive outcome of individual studies, with the whiskers corresponding to the 95% confidence interval (95% CI). The diamonds correspond to the study’s positive outcome and 95% CI of every subgroup and overall.

Figure 2 shows the forest plot describing the overall outcome of PRP treatment vs. control (efficacy of PRP vs. no efficacy). In Figure 3, a second forest plot compares the outcomes evaluated in multiple investigations (ultrasonography, histology, biomolecular concentration) to verifying if heterogeneity varies between groups.

The graphs show no significant difference between the PRP group and the control group (Figure 2: OR 50.98; 95% CI 4.28–606.93; *p* = 0.002); (Figure 3: OR 32.46; 95% CI 3.43–306.68; *p* = 0.002). Moreover, both graphs show a high heterogeneity of results (Figure 2: I^2^ = 85%; *p* < 0.00001. Figure 3: I^2^ = 84%; *p* < 0.00001), confirming that involved studies differ from each other and standardization of the process is lacking.

## 4. Discussion

In the last two decades, the use of blood derivates for treating tenodesmic lesions has been increasing. As in human medicine, in the veterinary field, the most popular blood-product is PRP. In clinical practice, it is assumed that this blood concentrate promotes the healing process and reduces inflammation [56,57,58,59,60], but there is no agreement between researchers about this issue. Although most of the studies evaluated in this systematic review endorse PRP as an efficient treatment for tendon and ligament injuries, meta-analysis results do not confirm these conclusions due to the high risk of bias and the high heterogeneity. Indeed, a great number of uncontrolled or biased trials about PRP efficacy only suggest, but do not demonstrate, the beneficial effects of PRP [42,50,52,61]. The rigor of most clinical studies is weakened by the absence of a control group and the omission of histological evaluation. It would be unethical and could compromise welfare to treat an injured horse with a placebo [62], and economic constraints can limit the capacity to send sample tissues for histological examination. Therefore, the number of horses with owner consent for a controlled clinical trial is generally limited, and treatment and control groups lack high homogeneity [36].

**Figure 4:**
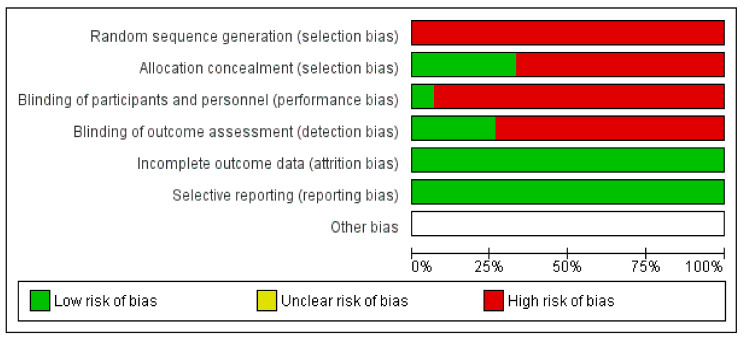
Risk of bias.

**Figure 5:**
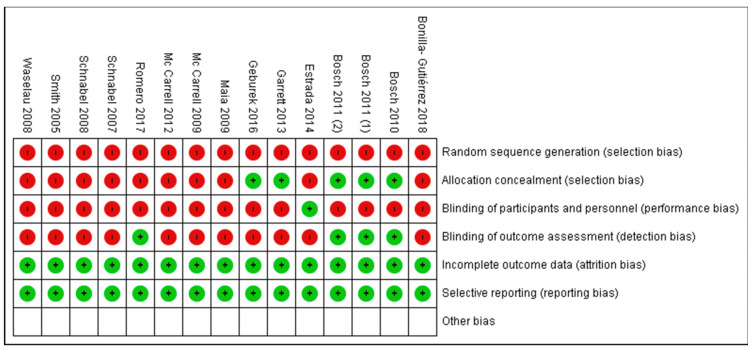
Summary of risk of bias.

The studies’ methods were dissatisfied in most of the selected trials. The quality was inversely correlated with PRP performance in clinical trials; low-quality study design, with a high risk of bias, was most of the time associated with a positive performance of PRP. There were no studies with a low/moderate risk of bias in the systematic review and meta-analysis, as shown in Figure 4 and Figure 5.

The decision of including studies with a high risk of bias in the present systematic review and meta-analysis certainly increases the heterogeneity of the sample [63] but, on the other hand, also allows to establish a relationship between the design and the outcome of the study, as already suggested by Brossi et al. [15].

Only three trials included in the systematic review were RCTs, and one of these [35] (study ID in Table 1: 10) affirms that there was no evidence that PRP promotes the healing process. In this case, a rigorous methodology in the trial may be related to poor PRP performance [47].

Differently from recent human and veterinary reviews [15,22,23,25,26,27,64], both in vivo and in vitro investigations were included in this study. Tendon and ligament explants cultured in PRP showed enhanced expression of matrix protein and growth factors with no concomitant increase in the catabolic molecules. These findings support in vivo investigations of PRP as an autologous treatment for tendon and ligament injuries [39,40].

The hypothesis was that in vitro studies have a better standardization of the methods and more objective outcomes. Indeed, in vitro trials provide a controlled environment of all the experiments [65], even though investigations were often carried out on healthy tendons and ligaments, which probably have a different response to PRP than injured ones [66]. Furthermore, all selected studies had a high risk of bias due to the absence of the operators’ blinding. It was shown that in vitro PRP studies yield better results than clinical trials do, which indicates that experimental settings cannot perfectly mimic the conditions of natural tendon repair [15,33]. Thus, our hypothesis that including in vitro studies in the meta-analysis would have reduced the level of bias and increased the significance of the analysis was not confirmed.

There are many flaws in the selected trials: first, the absence of a control group. The control group is essential to isolate the independent variable’s impact on the dependent variable, identifying different problems that can undermine the study’s credibility [67]. The meta-analysis was conducted only on fifteen of the twenty-four examined trials because they were the only ones with a control group.

Second, another important flaw in the evaluated studies was the extreme variability of the preparation methods and characteristics of the platelet concentrate. As shown in Table 2, PRP was obtained using different techniques, and the final product had a platelet concentration that varies between trials. To allow a better comparison between the studies, we try to include only investigations that reported the platelet concentration and PRP preparation protocol.

The platelet concentrate could be produced by centrifugation (single or double), filtration, or commercial kits; authors could also choose if they activated the product or not [31,46,68]. Some of the selected clinical studies produced PRP by double centrifugation. They obtained good platelet concentrations, although it was reported that the technique causes significant alterations in platelet morphology and that it is more sensitive to small errors during preparation. The highest thrombocyte concentrations were obtained by filtration [46,69]. With both techniques, the outcomes reported a beneficial PRP effect in treating tenodesmic lesions: therefore, further studies are needed to clarify which methodologies are the most appropriate.

Furthermore, PRP was used in different concentrations in the selected trials: it has been shown that a 10% PRP solution has a greater biological effect on suspensory ligament explants than a 5% solution [54], and this effect is still better at 100% usage [40]. However, various PRP concentrations reached in these studies were sufficient to influence tendons and ligaments healing positively. It is recognized that lower platelet concentrations have minimal effects on tendons/ligaments healing, while too high concentrations may have negative effects on the tissue repair process [70]. McLellan et al. [64] suggest that growth factor concentration and platelet count are positively correlated and that higher concentrations of PRP solution promote a greater healing response. For this reason, the ideal preparation method should have a high collection efficiency and degree of repeatability, as commercial kits warrant.

Another controversial topic in the preparation of the PRP is the necessity to activate the product or not. The three studies that activated the product [48,54,55] (study ID in Table 1: 12, 23, 24) also reported good performance of PRP in the treatment of tenodesmic lesions, while the two studies [35,47] (study ID in Table 1: 9, 10) that indicated the low efficacy of PRP did not proceed with activation; therefore, the question should be clarified through clinical and experimental studies.

The techniques of PRP administration were relatively similar: there was not a significant difference in the type of sedation and nerve blocks, the volume of product injected, the frequency of administration, and the materials used for injection between trials.

The absence of blinding was another limit in most studies. Figure 4 and Figure 5 show the risk of bias of the trials submitted to meta-analysis; blinding reduces bias in the studies’ outcome, so, if possible, practitioners evaluating the results should not be aware of the treatment. There is strong evidence that the absence of blinding produces exaggerated conclusions on the treatment’s effect, especially when evaluating subjective outcomes (pain, lameness, pleasure, etc.) [71]. In addition to the blinding of the outcome assessor, all the trial participants should not be aware of the assigned intervention: animals were certainly unaware of the treatment they were subjected to, but most of the time, operators know what they were injecting in the lesion. Placebo (usually saline solution) and PRP have a different appearance, evident to the operators injecting into the lesion [35].

Another flaw was the low number of patients involved in the trials. Studies that enrolled a larger number of horses [35,52] (study ID in Table 1: 10, 18) gave different opinions on the usefulness of PRP in the treatment of tenodesmic injuries, so we cannot correlate negative or positive effects to the size of the sample, but studies performed on few participants may be subject to type II errors.

The difference in the characteristics (age, sex, breed, and aptitude) of selected horses is another problem that does not allow to standardize PRP preparation because there is strong evidence that PRP is affected by intrinsic factors such as the breed, gender, and age [68]. Furthermore, the aptitude of the horse affected the outcome when this was evaluated as performance.

Selected clinical trials used PRP in acute, chronic, and experimentally induced tenodesmic lesions. Digital superficial flexor tendon (DSFT) and suspensory ligament (SL) of the fetlock were involved in different areas. Platelet-rich plasma gave unsatisfactory results in both SL [35] (study ID in Table 1: 10) and SDFT trials [47] (study ID in Table 1: 9). The tissue heterogeneity and the different stages of the disease do not provide precise PRP use recommendations based on the tissue damage [72]. Platelet-rich plasma should be used in acute lesions because there is only limited evidence that its injections are beneficial in chronic injuries [22].

The definition of the outcomes evaluated was flawless; the ways of presenting the outcomes, even though very different between them, were always objective and included: pain scales, concentration of growth factors, DNA, GAGs, pyrrole and collagen, evaluation of the elastic modulus of the treated tendons, and validated evaluation of the performance.

Most of the clinical trials suggested the use of a standardized rehabilitation/training program, which is a fundamental part for the complete resolution of tenodesmic injuries [73]. The exercise plan aims to provide an ascending regimen of activity that optimizes scar tissue function without causing damage to the tendon/ligament [74]. Exercise can be seen as a factor that, when used in combination with PRP treatment, improves healing of tendons and ligaments but can make it difficult to recognize if the outcomes depended only on PRP or the combination of those two elements.

To reduce the heterogeneity of the studies, a further meta-analysis of the subgroups sharing the same outcome was performed. Even so, this review did not show strong evidence that PRP improves the healing of tendon and ligament injuries in the horse, and there is currently no statistical basis for evaluating the effectiveness of PRP in clinical practice.

## 5. Conclusions

Although several studies seem to demonstrate that PRP has a beneficial effect on tendon and ligament healing without significant adverse effects; similar to previous studies, the present meta-analysis confirms that there is no definitive evidence of its effectiveness. Literature dedicated to the topic is quite extensive, but at the same time, it lacks standardization of study protocols, platelet separation techniques, and outcome measures.

The present review confirmed that poorly designed and biased studies that are not blinded or controlled and adopt inadequate outcome measures favored positive results.

In the near future, we suggest conducting studies that overcome those problems to obtain outcomes that might clarify this issue.

## Figures and Tables

**Table 1:** Different sources of bias present in the studies included in the systematic review.

Study ID	Study	Random Sequence Generation (Selection Bias)	Allocation Concealment (Selection Bias)	Blinding of Participants and Personnel (Performance Bias)	Blinding of Outcome Assessment (Detection Bias)	Incomplete Outcome Data (Attrition Bias)	Selective Reporting (Reporting Bias)	Risk of Bias
1	Argüelles, 2008	−	−	−	−	+	+	↑
2	Bazzano, 2013	−	−	−	−	+	+	↑
3	Bonilla–Gutiérrez, 2018	−	−	−	−	+	+	↑
4	Bosch,2010	−	+	−	+	+	+	↓
5	Bosch, 2011 (1)	−	+	−	+	+	+	↓
6	Bosch, 2011 (2)	−	+	−	+	+	+	↓
7	Boswell, 2014	−	−	−	−	+	+	↓
8	Castelijns, 2011	−	−	−	−	+	+	↑
9	Estrada, 2014	−	−	+	−	+	+	↓
10	Garrett, 2013	−	+	−	−	+	+	↓
11	Geburek, 2016	−	+	−	−	+	+	↓
12	Maia, 2009	−	−	−	−	+	+	↑
13	McCarrell, 2009	−	−	−	−	+	+	↑
14	McCarrell, 2012	−	−	−	−	+	+	↑
15	Rindermann, 2010	−	−	−	−	+	+	↑
16	Romagnoli, 2015	−	−	−	−	+	+	↑
17	Romero, 2017	−	−	−	+	+	+	↓
18	Scala, 2014	−	−	−	−	−	+	↑
19	Spadari, 2010	−	−	−	−	+	+	↑
20	Schnabel, 2007	−	−	−	−	+	+	↑
21	Schnabel, 2008	−	−	−	−	+	+	↑
22	Smith, 2006	−	−	−	−	+	+	↑
23	Waselau, 2008	−	−	−	−	+	+	↑
24	Zuffova, 2013	−	−	−	−	+	+	↑

(+) source of bias; (−) absent source of bias; (↑) high risk of bias; (↓) low risk of bias.

**Table 2:** Techniques used for platelet-rich plasma (PRP) preparation and final platelet concentration in the studies included in the systematic review. In bold the studies included in the meta-analysis.

Study	Commercial System	Centrifugation	Filtration	Platelet Concentration
Arguelles, 2008		√		250 × 10^6^ platelets/mL
Bazzano, 2013		√		540.000 ± 98.000 platelets/µL
Bonilla-Gutierrez, 2018		√		N.D
**Bosch, 2010**	√			639.7 ± 103.2 × 10^9^ platelets/L
**Bosch, 2011 (1)**	√			N.D
Bosch, 2011 (2)	√			3.8 × hematic concentration
Boswell, 2014		√		236.750 ± 35.714 platelets/µL
Castelijns, 2011			√	850 ± 244 × 10^9^ platelets/L
**Estrada, 2014**	√			162.0 ± 43.6 × 10^3^ platelets/µL
**Garret, 2013**	√			966.000 ± 189.000 platelets/µL
**Geburek, 2016**		√		892.37 ± 364.7 × 10^3^ platelets/µL
**Maia, 2009**		√		407.500 ± 58.800 platelets/µL
**McCarrell, 2009**	√			1 × 10^9^ platelets/mL
McCarrell, 2012	√			N.D
Rindermann, 2010	√			160–197 × 10^9^ platelets/L
Romagnoli, 2015		√		1045 × 10^3^ platelets/µL
Romero, 2017		√		263.3 × 10^3^ ± 99.9 × 10^3^ platelets/µL
Scala, 2014		√		1 × 10^6^ platelets/µL
**Schnabel, 2007**	√			395 × 10^3^ platelets/µL
Schnabel, 2008	√			N.D
**Smith, 2006**		√ *		520.000 platelets/µL
Spadari, 2010		√		7–11 × hematic concentration
**Waselau, 2008**	√			1.37·10^6^ ± 1.11 × 10^4^ platelets/µL
Zuffova, 2013		√ *		466.5 × 10^9^ platelets /L

(√) technique used for PRP preparation; (*) double centrifugation; (N.D) value not declared.

**Table 3:** Type of study, sample constitution, interventions applied, and outcomes considered in the studies involved in the systematic review. In bold: studies included in the meta-analysis.

Study	Type of Study	Sample	Interventions	Outcomes
Argüelles, 2008	No-RCTs	Two horses with tendinopathy of SDFT and three horses with desmitis of SL.	Injection of 5–8 mL of PRP into the lesion.	Improvements in the ultrasonographic aspect of the lesions, especially in SDFT tendonitis, decreased degree of lameness and response to flexion test. All horses returned to their preinjury level of performance.
Bazzano, 2013	No-RCTs	Fifteen horses affected by tendinitis of SDFT or DDFT.	Injection of 0.5–5 mL of PRP into the lesion.	The ultrasonographic aspect of tendons, 50 days after the PRP treatment, was comparable to healthy tendons; all horses showed clinical improvement. All the patients returned to train and compete; no reinjury occurred within 12 months from the beginning of the treatment.
**Bonilla–Gutiérrez, 2018**	RCTs	SL and SDFT samples from six horses.	Four SL and 50% of concentration. One SL and 1 SDFT did not receive any treatment and were used as a control group.	The concentration of IL-1β, TNF-α, IL-4, IL-1 receptor antagonist, PDGF- ββ, TGF-β1, and HA released from incubated tendons and ligaments was higher in the PRP group than in the control group.
**Bosch, 2010**	No-RCTs	Six horses with induced lesions of the SDFT in both forelimbs.	Injection of 3 mL of PRP in the lesion and 3 mL of saline solution in the other limb (placebo group).	The concentration of collagen, GAGs, and number of cells was higher in the PRP-treated tendons. The repair tissue in the PRP group showed a higher elastic modulus and breaking strength. Histologically, the PRP-treated tendons had a better organization of the collagen network and signs of increased metabolic activity.
**Bosch, 2011 (1)**	No-RCTs	Six horses with induced lesions of the SDFT in both forelimbs.	Injection of 3 mL of PRP in the lesion and 3 mL of saline solution in the other limb (placebo group).	Blood flow, evaluated with CFD, was significantly higher in the PRP-treated group. The total number of blood vessels 24 weeks after lesion induction, determined with the Factor VIII staining, was significantly higher in the PRP-treated tendons.
**Bosch, 2011 (2)**	No-RCTs	Six horses with induced lesions of the SDFT in both forelimbs.	Injection of 3 mL of PRP in the lesion and 3 mL of saline solution in the other limb (placebo group).	The ultrasonographic tissue characterization showed an E-value significantly higher in PRP-treated tendons than the controls at weeks 2, 3, 5 and 8; the C-value in the PRP group became significantly higher from week 12. The B-value was very similar throughout the experiment for both groups. These results suggest that the PRP treatment accelerates the collagenous matrix organization into tendon bundles and their arrangement along the stress lines.
Boswell, 2014	CLS	Eight horses.	Explants of SDFT cultured in LR-PRP or placebo for 3 days.	In the LR-PRP group, the concentration of PDGF-ββ, TGF- β1, COL1A1, and COMP was increased. The concentration of IL-1 β, MMP-3, and MMP-13, instead was decreased.
Castelijns, 2011	No-RCTs	Eleven horses with desmitis of SL.	2.5 mL of platelet concentrate was injected into the lesion.	The lameness score showed a reduction in all of the treated patients. The ultrasonographic exam performed three months after the treatment showed a complete resolution of the lesion for 10 horses. Five horses returned to their previous level of work.
**Estrada, 2014**	No-RCTs	Eight horses with induced lesions of the SDFT of both fore- and hindlimbs.	Injection of 2.5 mL of PRP or saline solution into the lesion (placebo group). The treatment assignment was randomized, and the operator was not aware of what he was injecting.	The PRP-treated tendons presented a significantly lower concentration of GAGs when compared to the control group. Other compositional, biomechanical, ultrasonographic, and histological parameters showed no significant differences.
**Garrett, 2013**	RCTs	Thirty-nine horses with proximal sesamoid bone inflammation and SL-branch desmitis.	Injection of 3 mL of PRP or saline (placebo group) in the affected SL- branch or proximal sesamoid bone junction.	Considering the number of races started and earnings in the following 2, 3, and 4 years, most of the horses treated with PRP started at least one race during the 2nd racing year. No significant differences were found between groups regarding starts during the 3rd and 4th year of competition and earnings.
**Geburek, 2016**	RCTs	Twenty horses with tendinitis of the SDFT of one or both forelimbs.	Injection of 3 mL of PRP or saline solution (placebo group) in the lesion.	Lameness decreased significantly at 8 weeks in the PRP-treated group, at 12 weeks in the placebo group. Ultrasonographically, there were no differences in the cross-sectional area between the two groups; 80% of the PRP-treated horses reached their previous or a higher performance level after 12 months compared to 50% in the control group. After 24 months, these proportions were 60% and 50%, respectively.
**Maia, 2009**	No-RCTs	Six horses with induced lesions of the SDFT in both forelimbs.	2.5 mL of activated PRP was injected in the right forelimb, 2.5 mL of saline solution was administered in the left forelimb (placebo group).	Thirty-six days after induced lesions, the histologic exam showed that injuries under PRP treatment presented a more uniform and organized tissue repair than the placebo group.
**McCarrell, 2009**	CLS	Five horses.	Tendons and ligaments cells from treated horses were cultured with PRP and placebo.	The PRP group has a higher concentration of TGF-b1, PDGF-BB, COL1A1, COL3A1, COMP, and a lower expression of MMP-13.
**McCarrell, 2012**	CLS	Eight horses.	Tendons were chopped into explants and placed into culture plates; then, they were cultured with standard PRP, high-concentration PRP, leukocyte-reduced PRP, concentrated-leukocyte PRP, or placebo.	The expression of COMP and COL1A1/COL3A1 ratio was increased in the PRP groups, while the expression of MMP-13 was decreased.
Rindermann, 2010	Case report	Seven horses with tendinitis of SDFT, DDFT or with desmitis of inferior check ligament.	Injection of 2–4 mL of ACP into the lesion.	All horses treated with platelet concentrate returned to their previous level of work; the ultrasonographic aspect of tendons/ligaments improved.
Romagnoli, 2015	No- RCTs	Twenty horses with desmitis of SL.	Injection of 0.8–4 mL of PRP into the lesion.	Twenty-four months after the treatment, sixteen horses returned to their previous activity, while four animals recidivated in different regions of SL.
**Romero, 2017**	No-RCTs	Twenty horses with induced lesions in SDFT in both forelimbs.	Horses received 7 mL of PRP or 7 mL Ringer’s lactate solution (placebo group) in assigned tendons 1 week after the injury induction.	In the PRP-group, ten weeks after the treatment, there was a significant reduction in FPS, CSA, and TES. Furthermore, PRP-treatment was associated with a better histopathological outcome.
Scala, 2014	No- RCTs	Ninety-nine horses with tenodesmic lesions.	Injection of PRP into the lesion (the amount of injected product varies depending on the size of the lesion).	Complete clinical and ultrasonographic healing was obtained in 81% of treated horses; 12% had an improvement and 7% a failure.
**Schnabel, 2007**	CLS	Six horses with induced lesions in SDFT in both forelimbs.	Cultures were established by 5 tendon explants (two replicates/group); culture media were whole blood, plasma, PRP, PPP, and bone marrow aspirate at different concentrations.	Tendons cultured in PRP showed enhanced COL1A1, COL3A1, and COMP concentrations, with no increase in the catabolic molecules like MMP-3 and MMP-13.
**Schnabel, 2008**	CLS	Six horses.	Tendons were chopped into explants; cultures were established with five explants/well of six-well plates with two replicates/treatment group (defined by culture medium)/horse. Culture media were whole blood, plasma, PRP, PPP, bone marrow aspirate at different concentrations.	Tendons cultured in PRP showed an enhanced concentration of COL1A1, COL3A1, COMP, decorin, with no concomitant increase in the catabolic molecules such as MMP-3 and MMP-13.
**Smith, 2006**	CLS	Five horses.	Cells from SL sections were recollected and cultured with acellular bone marrow, PRP, equine serum, fetal bovine serum, and medium (placebo group).	There was an increase in mean COMP production and mean H-Leucine incorporation in ligaments treated with PRP.
Spadari, 2010	No- RCTs	Ten horses with desmitis of SL.	Injection of PRP into the lesion.	Nine horses showed an improvement in the ultrasonographic aspect of the SL and returned to the activity.
**Waselau, 2008**	No-RCTs	Nine horses.	Injection of 3 mL of PRP into the lesion.	The number of starts and earnings during the first and third year was lower than earnings and starts during the year before the injury. During the second year, the number of starts and earnings was higher.
Zuffova, 2013	No-RCTs	Twenty-two horses with tendinitis of SDFT.	Injection of PRP into the lesion (the amount of injected product varies depending on the size of the lesion).	Horses with acute lesions ran 56% of races in the follow-up period, while animals with chronic injuries ran 30% of competitions.

ACP: autologous conditioned plasma; CFD: color flow Doppler; CLS: controlled laboratory study; COL1A1: alpha-1 type I collagen; COL3A1: alpha-1 type III collagen; COMP: cartilage oligomeric matrix protein; CSA: cross-sectional area; FPS: fibre pattern score; GAGs: glycosaminoglycans; HA: hyaluronic acid; IL-1α: interleukin-1α; IL-1β: interleukin-1β; IL-4: interleukin-4; LR-PRP: leukocyte-rich platelet-rich plasma; MMP-13: matrix metalloproteinase-13; MMP-3: matrix metalloproteinase-3; No-RCTs: not randomized controlled trial; PDGF- ββ: platelet-derived growth factor-ββ; PPP: platelet-poor plasma; RCT: randomized controlled trial; SDFT: superficial digital flexor tendon; SL: suspensory ligament; TES: tendon echogenicity score; TGF-β1: Transforming growth factor-1β; TNF-α: Tumor necrosis factor-α.

## Data Availability

The data presented in this study are available on request from the corresponding author.
